# Editorial: Multi-target drug discovery: an opportunity for novel and repurposed bioactive compounds

**DOI:** 10.3389/fphar.2026.1963915

**Published:** 2026-08-26

**Authors:** Francisco Jaime Bezerra Mendonça-Junior

**Affiliations:** State University of Paraiba, Campina Grande, Brazil

**Keywords:** bioactive compounds, bioinformatics, complex diseases, drug repurposing, molecular docking, molecular dynamics simulations, multi-target drug discovery, secondary metabolites

## Introduction

The growing recognition that most human diseases arise from complex and interconnected molecular networks has challenged the traditional paradigm of “one drug-one target-one disease” that has dominated pharmacological research for decades. Multifactorial disorders, including cancer, neurodegenerative diseases, metabolic syndromes, psychiatric disorders, and chronic inflammatory conditions, involve dynamic interactions among numerous signaling pathways, often rendering single-target therapies insufficient to achieve durable therapeutic responses. Consequently, multi-target drug discovery (MTDD) has emerged as a promising strategy capable of simultaneously modulating multiple biological pathways, thereby enhancing therapeutic efficacy, reducing drug resistance, and improving clinical outcomes.

Recent advances in systems biology, computational chemistry, artificial intelligence (AI), network pharmacology, multi-omics technologies, and high-throughput screening have significantly accelerated the identification and characterization of multitarget therapeutic agents. Moreover, natural products and drug repurposing strategies have increasingly attracted attention due to their intrinsic polypharmacological properties and favorable translational potential. In this context, the Research Topic *“Multi-Target Drug Discovery: An Opportunity for Novel and Repurposed Bioactive Compounds”* was organized to provide a multidisciplinary forum highlighting recent advances in the discovery, characterization, optimization, and repurposing of multitarget therapeutic agents.

The articles included in this Research Topic collectively demonstrate the breadth and versatility of contemporary multitarget pharmacology, encompassing natural products, synthetic compounds, computational approaches, systems pharmacology, translational research, and experimental validation.

## Findings in this volume

Natural products remain among the most valuable sources of multitarget therapeutic agents. In this regard, Wang et al. investigated the therapeutic effects of Ramulus mori (Sangzhi) alkaloids in a dehydroepiandrosterone-induced rat model of polycystic ovary syndrome (PCOS). Their study demonstrated that Sangzhi alkaloids improve reproductive and metabolic dysfunction through the simultaneous modulation of gut microbiota composition, oxidative stress, inflammatory pathways, and metabolic homeostasis. These findings reinforce the concept that complex endocrine disorders may benefit from therapeutic interventions capable of targeting multiple biological systems simultaneously.

Similarly, Fakhri et al. explored the neuroprotective effects of *Ferula ammoniacum* gum aqueous extract in an aluminum chloride-induced model of Alzheimer’s disease. Their results revealed that the extract attenuated behavioral deficits, oxidative stress, neuroinflammation, and histopathological alterations, while evidence obtained using naloxone suggested the involvement of opioidergic signaling pathways. This work highlights the potential of natural multitarget interventions to simultaneously regulate several pathological processes involved in neurodegeneration.

The multifactorial nature of neurodegenerative disorders is further illustrated by the study of Ríos et al., who developed a series of conformationally constrained tetrahydroisoquinoline derivatives displaying dual acetylcholinesterase inhibitory and antioxidant activities. Through the integration of synthetic chemistry, enzymatic assays, molecular docking, and density functional theory calculations, the authors demonstrated that these compounds represent a novel multitarget-directed ligand scaffold capable of addressing both cholinergic dysfunction and oxidative stress in Alzheimer’s disease.

Complementing these experimental approaches, Al Hashmi et al. employed network pharmacology, molecular docking, molecular dynamics simulations, and ADMET prediction to investigate the therapeutic potential of *Lycii Fructus* compounds against Parkinson’s disease. Their study identified several bioactive molecules capable of interacting with key pathological targets, including AKT1, TNF, IL-6, IL-1β, and MAOB, thereby providing a systems-level perspective on the multitarget mechanisms underlying the neuroprotective effects of traditional medicinal compounds.

Cancer remains one of the most important therapeutic areas driving the development of multitarget therapeutics. In this Research Topic, Zhou et al. reported the identification of chalcone-containing small molecules capable of simultaneously targeting the PD-1/PD-L1 immune checkpoint pathway and tubulin polymerization. Their lead compound demonstrated dual biological activity and significant antitumor efficacy *in vivo*, illustrating how the integration of immunomodulatory and cytotoxic mechanisms within a single molecular scaffold may provide innovative strategies for cancer treatment.

Likewise, Zhang et al. adopted an integrative multi-omics strategy to elucidate the mechanisms underlying the antitumor effects of berberine in triple-negative breast cancer. Combining network pharmacology, transcriptomics, single-cell sequencing, molecular docking, molecular dynamics simulations, and experimental validation, the authors demonstrated that berberine simultaneously regulates multiple oncogenic pathways, including SRC/STAT3 and PI3K/AKT signaling, while modulating tumor stemness and the tumor immune microenvironment. Their findings exemplify the systems pharmacology paradigm increasingly adopted in modern oncology drug discovery.

The importance of natural compounds in oncology is further highlighted by the review article of Elsori et al., which comprehensively summarizes the anticancer potential of saponins against glioblastoma multiforme. The authors discuss how these natural compounds exert multitarget effects through the regulation of apoptosis, oxidative stress, cell cycle progression, proliferation, migration, and invasion pathways, emphasizing their potential as multifunctional therapeutic agents for highly aggressive brain tumors.

Beyond oncology and neurodegeneration, multitarget approaches are increasingly being explored for chronic fibrotic diseases. Cao et al. investigated the pharmacological mechanisms of *Polygonatum odoratum* in idiopathic pulmonary fibrosis using an integrated framework combining bioinformatics, network pharmacology, molecular modeling, and experimental validation. Their results identified several key targets, including EGFR, HIF1A, GSK3B, MTOR, and BCL2, demonstrating that phytochemicals can simultaneously modulate multiple fibrogenic pathways involved in disease progression.

Psychiatric disorders also represent an important frontier for multitarget pharmacology. In their comprehensive review, Rautela et al. examined the therapeutic potential of phosphodiesterase PDE1B and PDE10A inhibition for schizophrenia treatment. By integrating medicinal chemistry, structural biology, pharmacology, and clinical evidence, the authors highlighted the possibility of simultaneously modulating positive, negative, and cognitive symptoms through the coordinated regulation of cyclic nucleotide signaling pathways. Furthermore, they emphasized the emerging potential of dual PDE1B/PDE10A inhibitors as next-generation antipsychotic agents.

The concept of translational repurposing is exemplified by the contribution of Rossini et al., who reviewed the pharmacological trajectory of phosphoethanolamine from endogenous metabolite to therapeutic candidate. Their analysis revealed how mechanistic studies identifying succinate dehydrogenase inhibition have expanded the therapeutic potential of phosphoethanolamine beyond its initial applications, opening new opportunities for diseases associated with mitochondrial dysfunction and ischemia-reperfusion injury.

Further expanding the translational landscape of this Research Topic, Sharif et al. investigated the therapeutic potential of two complementary lipid-modulating strategies to address the complex interplay between metabolic dysfunction, amyloid biology, and neurovascular impairment in type 2 diabetes. Using a preclinical model, the authors demonstrated that a second-generation probucol analogue (HA1) and APOC3 siRNA differentially modulate interconnected pathways involved in lipoprotein metabolism, amyloid-β homeostasis, neuroinflammation, and blood–brain barrier integrity. Their findings further illustrate how the repurposing and optimization of established pharmacological scaffolds can generate multitarget therapeutic interventions capable of simultaneously modulating multiple disease-driving mechanisms, reinforcing the translational potential of polypharmacology for complex multifactorial disorders.

Finally, Biju and Osborne provide a comprehensive review addressing bacterial endophytic secondary metabolites as a source of novel bioactive compounds. Their contribution presents an integrated roadmap encompassing microbial isolation, metabolite production, purification, structural characterization, pharmacokinetic and toxicological evaluation, and artificial intelligence-assisted drug discovery approaches. Particularly noteworthy is their discussion of anti-virulence strategies targeting bacterial pathogenicity islands, which represents a paradigm shift from conventional antimicrobial approaches toward multitarget modulation of pathogenic mechanisms.

## Conclusions and future outlook

Collectively, the studies assembled in this Research Topic underscore several important trends shaping the future of pharmacological research. First, natural products continue to represent a rich source of structurally diverse multitarget compounds. Second, the integration of computational methods, systems biology, and experimental pharmacology has become indispensable for understanding complex therapeutic mechanisms. Third, drug repurposing and translational pharmacology offer efficient pathways for accelerating therapeutic innovation. Finally, the convergence of artificial intelligence, multi-omics technologies, and network pharmacology is transforming the discovery and optimization of multitarget therapeutics.

Although considerable challenges remain, including target selectivity optimization, pharmacokinetic profiling, safety assessment, and clinical translation, the studies presented in this collection clearly demonstrate that embracing biological complexity rather than simplifying it may provide the key to developing more effective therapies for multifactorial diseases.

We sincerely thank all authors for their valuable contributions, the reviewers for their insightful evaluations and constructive suggestions, and ALL the editorial team of *Frontiers in Pharmacology*, including all the guest editor, for their continuous support throughout the development of this Research Topic. We hope that this collection will stimulate further interdisciplinary research efforts and contribute to advancing the discovery, development, and clinical translation of novel and repurposed multitarget bioactive compounds.

